# Lithium-Induced Sinoatrial Node Dysfunction

**DOI:** 10.7759/cureus.16778

**Published:** 2021-07-31

**Authors:** Ashish Sarangi, Sana Javed, Tanya Paul, Wail Amor

**Affiliations:** 1 Psychiatry, Texas Tech University Health Sciences Center, Lubbock, USA; 2 Psychiatry, Nishtar Medical University, Multan, PAK; 3 Psychiatry and Behavioral Sciences, Avalon University School of Medicine, Youngstown, USA

**Keywords:** lithium, bipolar disorder, sinoatrial node dysfunction, pacemaker, lithium toxicity, lithium-induced bradycardia

## Abstract

Lithium is a common mood-stabilizing drug for manic patients. We describe a case of sinoatrial node dysfunction in a patient with serum lithium levels within the therapeutic range. Given the symptomology and severity of the patient’s illness, after placing a permanent pacemaker, the patient was discharged on the preadmission dose of lithium.

## Introduction

Bipolar disorder is a highly disruptive mental health condition that can lead to impairment in the overall health and functioning of an individual [1]. Lithium (Li) is used as a first-line treatment in bipolar disorder and has been the mainstay of treatment for acute manic episodes, suicide prevention and prophylactic treatment for more than six decades [1,2]. Li is infamous for its narrow therapeutic range of 0.6-1.2mmol/L, with levels > 1.5mmol/L considered toxic [1]. Li is well absorbed in the intestinal tract, remains unchanged, and is renally excreted [1]. Therefore, the modified volume of distribution (age and weight), renal clearance, and drug-drug interactions severely affect the pharmacokinetics and bioavailability of Li [1].

Some of the common side effects of lithium include gastrointestinal disturbances such as nausea, vomiting, diarrhea, constipation. While others such as fine tremors, hypothyroidism, diabetes insipidus, and weight gain are also not uncommon [3,4]. The cardiotoxic effects of Li including the electrocardiographic changes may occur at therapeutic levels as well as toxic levels, such as sinus node dysfunction, sinoatrial block, intraventricular - and atrioventricular - conduction delay, ST depressions/elevations, T-wave depression, the Brugada syndrome, and QT wave changes [5,6].

In such patients, it is imperative to discontinue Li in order to improve bradycardia. However, some patients will require continuation of Li therapy, if refractory to alternative agents. The placement of a permanent pacemaker is vital to decrease symptoms in such patients [7].

We present a case of lithium-induced SA nodal dysfunction in a patient with bipolar disorder. We will discuss the mechanisms underlying the condition, present optimal solutions, and highlight the significance of understanding the pharmacokinetics of Li in such patients.

## Case presentation

**Figure 1:**
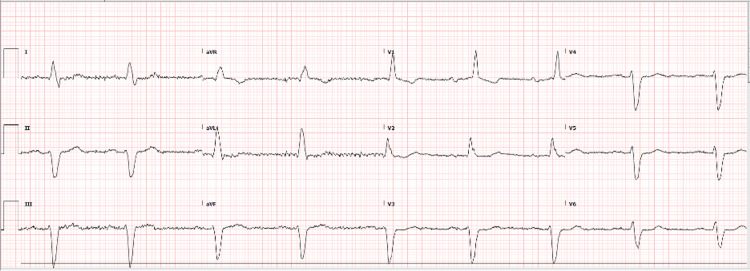
Initial ECG: Sinus bradycardia with prolonged PR interval, right bundle branch block, left anterior fascicular block, possible anterior myocardial infarction (probably old).

A 74-year-old male (Mr. A) with a known history of bipolar type 1 disorder, gout, and benign prostate hypertrophy presented to the emergency room with dizziness for one day. The patient was admitted to the hospital for symptomatic bradycardia with a heart rate of 52/min and blood pressure of 86/52mmHg. His ECG revealed sinus bradycardia with a prolonged PR interval as shown in Figure 1.

**Figure 2:**
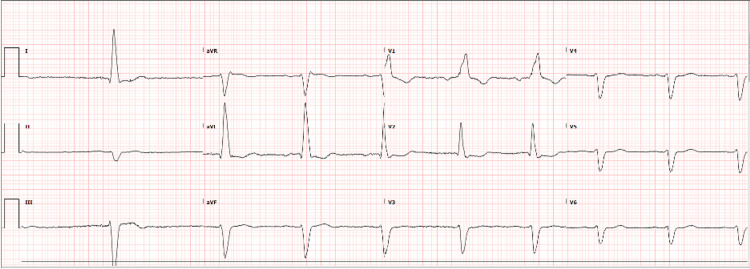
Patient’s ECG: Sinus bradycardia with marked sinus arrhythmia, right bundle branch block, left ventricular hypertrophy and ST-T change, possible anterior myocardial infarction (probably old), inferior myocardial infarction.

ECG was repeated two days later and revealed sinus bradycardia with marked sinus arrhythmia (Figure 2).

Transthoracic echocardiogram (TTE) showed a left ventricle ejection fraction estimated 60% to 64% with no regional wall motion abnormality. The diastole pattern was normal for age. He was also noted to have moderate aortic sclerosis, mild aortic stenosis, and trace aortic regurgitation. For the last five years, the patient has been taking lithium orotate 20mg daily (much lower than the therapeutic prescription of lithium carbonate: 112-225mg/day). Two months prior to the admission, the patient was referred to cardiology due to asymptomatic bradycardia and an abnormal ECG, where he was diagnosed with chronic conduction system disorder. The patient was placed on an event monitor due to bradycardia, and sinus pauses were noted. His medications included lisinopril 10mg, indomethacin 50mg PO TID, terazosin 2mg PO BID, furosemide 20mg PO daily, metoprolol 50mg PO BID, aspirin 81mg PO daily, and vitamin C supplement 1,000mg daily. His blood lithium level was 0.6mmol/L. The pacemaker was placed during the same hospitalization. Additional diagnosis of chronic kidney disease, stage III was made. Renal function test showed blood urea nitrogen (BUN) 20 (reference range: 7-20mg/dL), creatinine 1.3 (reference range: 0.84-1.21mg/dL), glomerular filtration rate (GFR) 54 (reference range: 90-120mL/min/1.73m^2^), and creatinine clearance 68 (reference range: 97-137mL/min). Mr. A had no personal or family history of cardiovascular disease, syncope, or sudden cardiac death. In March 2020, Mr. A's lithium orotate was decreased to 16 tablets a day as his creatinine was slightly elevated at 1.3, and he remained stable, no suicidal ideation was reported. Given the high likelihood of a reoccurrence of the bradyarrhythmia, a rate-modulated ventricular pacemaker was implanted. We discharged the patient on his preadmission dose of lithium orotate. His last cardiology follow-up was in May 2021, when the patient was asymptomatic.

## Discussion

Lithium was initially used for mania in 1949 but was withheld due to concerns of the drug adversely affecting cardiac patients [7]. Sinus node dysfunction from lithium use at therapeutic and toxic doses in both adults and children has been recorded [7]. The therapeutic level of lithium is in plasma levels between 0.6 and 1.0mmol/L [8]. Some lithium-related illnesses are right bundle branch block, left anterior hemiblock, atrioventricular block (AV) block, and sinus node dysfunction [7]. The reason for bradyarrhythmias in some cardiac patients is believed to be from hypercalcemia and hypothyroidism [7].

Lithium causes a blockage of the channels that control the cardiac electrical impulses and the sinus nodal pacemaker, and these are the myocyte voltage-gated sodium channels. Here, lithium would cause a downward shift in the level of intracellular potassium, which leads to a decrease in the conduction pathway and depolarization [9]. Another reason for bradycardia could be that the sinus node is affected because of lithium action on hyperpolarization-activated cyclic nucleotide-gated channels, L-type calcium channels, acetylcholine-gated potassium channels, and the sodium-calcium exchanger all of which control the sinus node [9].

Both sinus node dysfunction and T-wave abnormalities are seen with lithium use at therapeutic levels [9]. In our patient, sinoatrial node dysfunction was seen from long-standing lithium therapy, and it was not advisable to discontinue his lithium therapy, so a ventricular pacemaker was implanted.

Table 1 shows some of the cases similar to ours, over the past 20 years.

**Table 1:** Cases of lithium-induced nodal dysfunction reported in the past 20 years.

Sr. No.	Age, Gender	Dosage (mg/day)	Duration	Serum lithium level (mM)	Arrhythmia	Treatment	Reference	Year
1	60s, M	1,200	N/A	3.3	Sinus bradycardia and non-specific interventricular conduction delay	1. Intravenous fluids, atropine, and glucagon - no improvement; 2. Dopamine infusion - no improvement; 3. Transcutaneous pacing - The patient’s lithium level improved to 1.6mEq/L and creatinine improved to 0.9mg/dL by hospital day 3.	[10]	2021
2	46 year, F	900	15 years	0.7	Sinus arrest with intermittent junctional escape	1. Lithium stopped; 2. Temporary pacemaker - Sinus-node function improved over the next three days	[11]	2013
3	30 year, M	900		0.94 and 0.81	Sinus bradycardia	1. Lithium and Olanzapine discontinued, the patient was observed; 2. Patient was discharged on sodium valproate 1,000mg/day and haloperidol 10mg/day	[12]	2011
4	64 year, M	1,200	11 years	0.72	Sinus node arrest with an idioventricular escape rhythm	N/A	[7]	2007
5	56 year, F	600-1,000	7 years	0.7- 1.1	Sinus sick syndrome with a pacemaker	1. Lithium discontinued- improvement; 2. The patient did not respond to Carbamazepine/Valproate for Mania, started back on Lithium-sinus sick syndrome reappeared; 3. Permanent cardiac pacemaker	[13]	2002
6	42 year, F	900	7 years	3.86	Sinus bradycardia with inverted T waves and prominent U waves	1. Lithium discontinued; 2. Temporary cardiac pacing; 3. Hemodialysis once daily for three days	[14]	2000

While rare, there were two similar patient cases recorded over the last two decades who both eventually needed a pacemaker. The first one was a 46-year-old female patient [11] who developed sinus node dysfunction that was taking 900mg lithium for 15 years whose serum lithium level was 0.7mmol/L, which is within the therapeutic range for lithium. She developed sinus arrest with intermittent junctional escape and lithium was discontinued. A temporary pacemaker was indicated in this patient and her sinus node function improved over the next three days. The second case was of a 56-year-old female [13] who was placed on varying doses of 600-1,000mg lithium for seven years and her serum lithium level was 0.7-1.1mmol/L. She developed sick sinus syndrome and lithium was discontinued. This patient was started on carbamazepine/valproate for her mania but responded poorly so had to be placed back on lithium, and consequently, her sick sinus syndrome reappeared. A permanent cardiac pacemaker was placed in this patient. Some cases of sinus node dysfunction can be reversed from lithium discontinuation, and some cases need a cardiac pacemaker if lithium is reintroduced back into the patient treatment regimen [15].

## Conclusions

Although rare, symptomatic sinus node dysfunction can occur even at therapeutic levels of lithium in certain patients. Li therapy is safe to start in all patients who do not have a history of signifying sinus node dysfunction. However, if a patient has a history of dizziness and/or syncope, it is vital to have a thorough cardiological examination before prescribing Li. Similarly, a cardiology examination and/or consult should be ordered if the patient develops symptoms while on Li therapy.
